# Global Gene Expression Analysis Identifies Age-Related Differences in Knee Joint Transcriptome during the Development of Post-Traumatic Osteoarthritis in Mice

**DOI:** 10.3390/ijms21010364

**Published:** 2020-01-06

**Authors:** Aimy Sebastian, Deepa K. Murugesh, Melanie E. Mendez, Nicholas R. Hum, Naiomy D. Rios-Arce, Jillian L. McCool, Blaine A. Christiansen, Gabriela G. Loots

**Affiliations:** 1 Physical and Life Sciences Directorate, Lawrence Livermore National Laboratories, Livermore, CA 94550, USA; sebastian4@llnl.gov (A.S.); murugesh2@llnl.gov (D.K.M.); mendez20@llnl.gov (M.E.M.); hum3@llnl.gov (N.R.H.); riosarce1@llnl.gov (N.D.R.-A.); mccool1@llnl.gov (J.L.M.); 2 Molecular and Cell Biology, School of Natural Sciences, UC Merced, Merced, CA 95343, USA; 3 Department of Orthopedic Surgery, UC Davis Medical Center, Sacramento, CA 95101, USA; bchristiansen@ucdavis.edu

**Keywords:** osteoarthritis, aging, PTOA, gene expression, RNA-seq, cartilage degeneration, scRNA-seq, chondrocytes

## Abstract

Aging and injury are two major risk factors for osteoarthritis (OA). Yet, very little is known about how aging and injury interact and contribute to OA pathogenesis. In the present study, we examined age- and injury-related molecular changes in mouse knee joints that could contribute to OA. Using RNA-seq, first we profiled the knee joint transcriptome of 10-week-old, 62-week-old, and 95-week-old mice and found that the expression of several inflammatory-response related genes increased as a result of aging, whereas the expression of several genes involved in cartilage metabolism decreased with age. To determine how aging impacts post-traumatic arthritis (PTOA) development, the right knee joints of 10-week-old and 62-week-old mice were injured using a non-invasive tibial compression injury model and injury-induced structural and molecular changes were assessed. At six-week post-injury, 62-week-old mice displayed significantly more cartilage degeneration and osteophyte formation compared with young mice. Although both age groups elicited similar transcriptional responses to injury, 62-week-old mice had higher activation of inflammatory cytokines than 10-week-old mice, whereas cartilage/bone metabolism genes had higher expression in 10-week-old mice, suggesting that the differential expression of these genes might contribute to the differences in PTOA severity observed between these age groups.

## 1. Introduction

Osteoarthritis (OA) is a degenerative joint disease characterized by progressive cartilage loss, bone remodeling, synovial inflammation, and significant joint pain, often resulting in disability [1]. According to Center for Disease Control statistics, approximately 30 million people in the United States are estimated to have OA. Currently, there are no approved therapies available to prevent degeneration or rebuild articular cartilage destroyed by OA. Existing treatments mainly target the symptoms, and advanced OA often requires joint replacement [1]. A more in-depth understanding of the pathophysiology of the disease will likely lead to the development of novel therapeutic strategies for the prevention and/or treatment of OA.

Aging is a key risk factor for the development of OA. It is estimated that, by 2050, people over the age of 60 will account for more than 20% of the world’s population. Of that, ~15–20% will have symptomatic OA, and one-third of these people will be severely disabled. In addition to age, joint injury is also a common risk factor for OA. OA that develops because of a joint injury is defined as post-traumatic OA (PTOA). Clinical statistics reveal that ~50% of people with injury to the anterior cruciate ligament (ACL) or the meniscus will develop PTOA within 1–2 decades of the injury [2]. It has also been suggested that age and joint injury interact and have a combined effect on the development and progression of OA following joint trauma. The average time from meniscal injury to the onset of radiographic signs of OA in patients older than 30 was five years; in sharp contrast, patients between 17 and 30 years of age were asymptomatic for up to 15 years [3]. Although several studies have investigated the role of age and injury in the development of OA, the mechanisms by which age and injury interact to contribute to OA pathogenesis are still not well understood [4,5,6,7]. 

Recently, using a noninvasive tibial compression (TC) injury model of PTOA, we investigated molecular and structural changes in the knee joints of young (10-16 weeks-old) mice following ACL injury [8,9,10]. These mice showed significant cartilage erosion, subchondral bone loss, and osteophyte formation as early as 4–6 weeks post-injury [8,9,10]. Gene expression analysis using RNA-seq revealed that a significant number of genes associated with inflammatory responses were elevated shortly after the injury (one-day post-injury) and several genes associated with cartilage and bone remodeling were elevated at 1–6 weeks post-injury [8,10].

In the present study, we compared OA-associated changes in the knee joints of mice ranging in age from 10 to 95 weeks old, to determine the contribution of age to OA pathogenesis. First, we identified age-related molecular changes in healthy (uninjured) knee joints by comparing gene expression in 10-week-old, 62-week-old, and 95-week-old mice. Then, using the TC injury model [11], we compared molecular and structural changes associated with PTOA development between 10- and 62-week-old mice, to better understand how age and joint injury interact to contribute to the development of PTOA. We found that the old mice displayed a more severe cartilage degeneration phenotype and increased osteophyte formation in response to injury compared with the younger mice. We also identified significant differences in injury-induced response genes between old and young mice, including an increased expression of inflammatory-response related genes and reduced expression of genes involved in cartilage and bone development in old mice, which could have contributed to the more severe PTOA phenotype observed in old mice.

## 2. Results

### 2.1. Aging Up-Regulated Inflammatory Response-Related Genes and Down-Regulated Genes Associated with Cartilage Development and Homeostasis in Mouse Knee Joints

**Figure 1:**
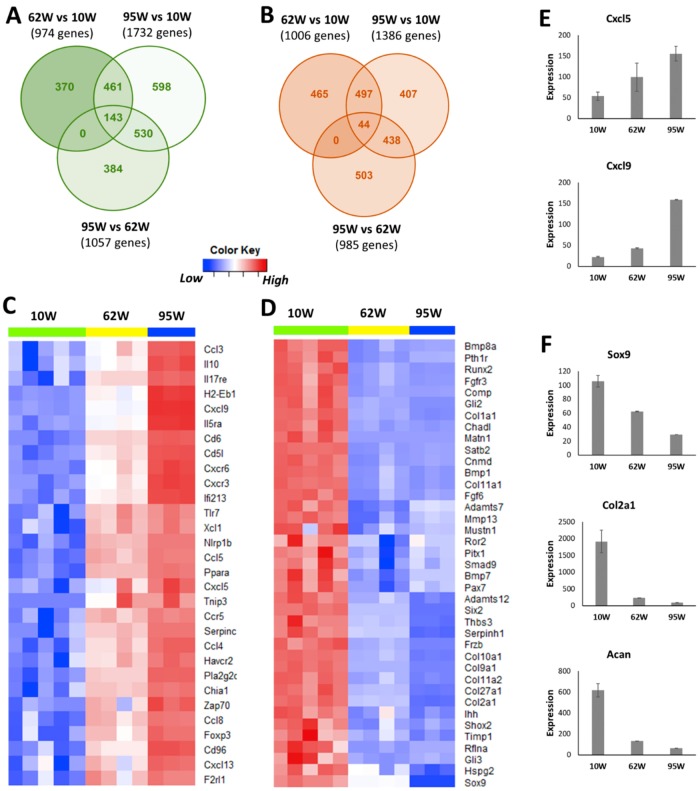
Age-related changes in the knee joint gene expression. Genes up- (**A**) and down-regulated (**B**) in 62-week-old (62W) mice and 95-week-old (95W) mice compared with 10-week-old (10W) mice and in 95-week-old mice compared with 62-week-old mice. (**C**) Inflammatory response-related genes up-regulated in both 62-week-old and 95-week-old compared with 10-week-old mice (top 30 genes). (**D**) Cartilage development-associated genes down-regulated in both 62-week-old and 95-week-old compared with 10-week-old mice. (**E**) Examples of inflammatory response genes showing progressive increase with age. (**F**) Key cartilage development-associated genes showing an age-related decrease in expression.

To determine aging-related molecular changes in healthy (uninjured) knee joints, we profiled the knee joint transcriptome of 10-week-old, 62-week-old, and 95-week-old mice using RNA-seq. Compared with 10-week-old mice, 974 and 1732 genes were up-regulated and 1006 and 1386 genes were down-regulated in 62-week-old mice and 95-week-old mice, respectively (Figure 1A,B, Table S1). Six-hundred and four genes including 42 arthritis-associated genes (*Mmp3*, *Ccl3*, *Ccr5*, *Csf3*, *Cxcr6*, *Lyz1*, *Il33*, and *Tnfsf15*, among others), 110 genes associated with immune response (*Tlr7*, *Csf3*, *Cd3d*, *Cd4*, *Nlrp1b*, and *Cd8a*, among others) and 47 inflammatory response-related genes (*Ccl5*, *Ccr5*, *Cxcl1*, and *Cxcl5*, among others) were up-regulated in both 62-week-old and 95-week-old old mice compared with 10-week-old mice (Figure S1A, Figure 1C). Of these common up-regulated genes, 143 genes including several key regulators of immune/inflammatory responses such as *Il5ra*, *Il17re*, *Cxcr3*, *Cxcr6*, *Cxcl5*, *Cxcl9*, *Ccl3*, and *Ccl4* had a significantly higher expression in 95-week-old mice compared with 62-week-old mice, suggesting a progressive increase in the levels of inflammatory mediators in the joint with age (Figure 1C,E). Genes down-regulated in both 62-week-old and 95-week-old mice compared with 10-week-old mice included 39 genes associated with cartilage development (*Col2a1*, *Col9a1*, *Sox9*, *Acan*, and *Comp*, among others), 40 genes associated with bone development (*Col1a1*, *Alpl*, *Sparc*, *Bglap*, and *Runx2*, among others), 35 genes involved in osteoblast differentiation, and 22 genes involved in chondrocyte differentiation (Figure 1D, Figure S1B–D). Several key regulators of cartilage development and homeostasis including *Sox9*, *Col2a1*, and *Acan* had the lowest expression in 95-week-old mice, indicating a reduction in cartilage anabolic responses with age (Figure 1F). 

### 2.2. Injury-Induced Knee Joint Degeneration was Accelerated in Old Mice

**Figure 2:**
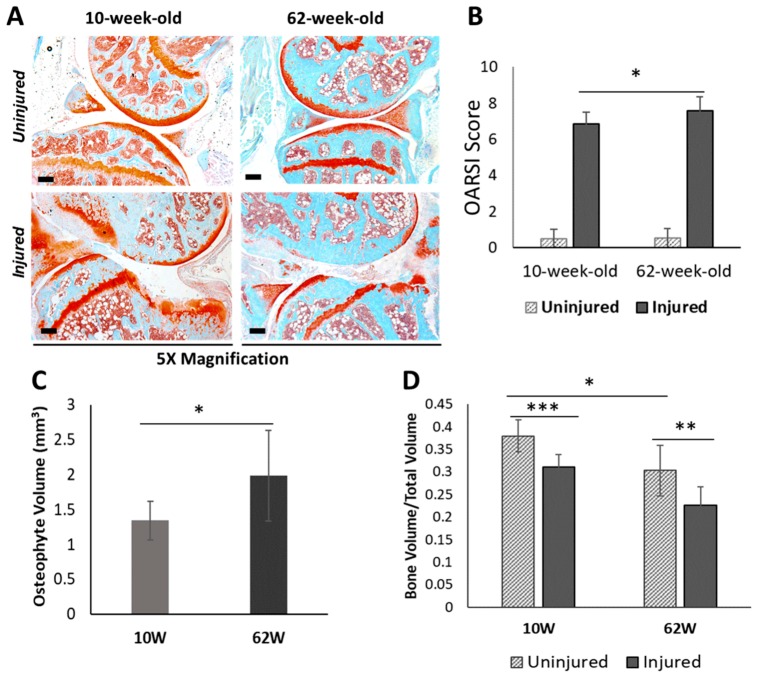
Characterization of post-traumatic osteoarthritis (PTOA)-associated structural changes in 10-week-old and 62-week-old mice. (**A**) Histological evaluation of uninjured contralateral joints and injured joints at six-week post-injury using Safranin-O and Fast Green staining which stains cartilage in red and surrounding tissue in green (5× magnification). Scale bars: 200 µm. (**B**) Osteoarthritis Research Society International (OARSI) scoring of histological sections of injured and uninjured contralateral joints at six-week post-injury. (**C**) Osteophyte volume at six-week post-injury. (**D**) Epiphyseal trabecular bone volume fraction (BV/TV) of the distal femur was quantified using µCT and analyzed between injured and uninjured contralateral joints at six-week post-injury. 10W: 10-week-old; 62W: 62-week-old. * *p* < 0.05, ** *p* < 0.01, *** *p* < 0.001.

To understand how aging impacts PTOA development after injury, we investigated structural changes in the knee joints of 62-week-old and 10-week-old mice six weeks after an ACL injury. Ten-week-old mice represent young adult humans whose cartilage is normally healthy, whereas 62-week-old mice represent a ~50–60 year-old human, an age group in which OA is prevalent. By six-week post-injury, both 10-week-old and 62-week-old mice exhibited severe cartilage degradation in the injured joints (Figure 2A,B). OA lesions were more severe in 62-week-old mice than in 10-week-old mice, where the majority of the femoral head was lacking the articular cartilage layer in the injured 62-week old mice (Figure 2A). Osteophyte formation was observed in both age groups by six-week post-injury, and 62-week-old mice had significantly more osteophytes than 10-week-old mice (Figure 2C). ACL injury also resulted in a significant reduction in subchondral bone volume in the femoral epiphysis in both age groups (Figure 2D). Older, 62-week-old mice displayed a significantly lower subchondral bone volume than 10-week-old mice before injury, and they also lost more subchondral bone (25% loss) than younger mice (18% loss) by six weeks after injury (Figure 2D).

### 2.3. Age-Related Differences in ACL Injury-Induced Gene Expression Changes in the Knee Joints

**Figure 3:**
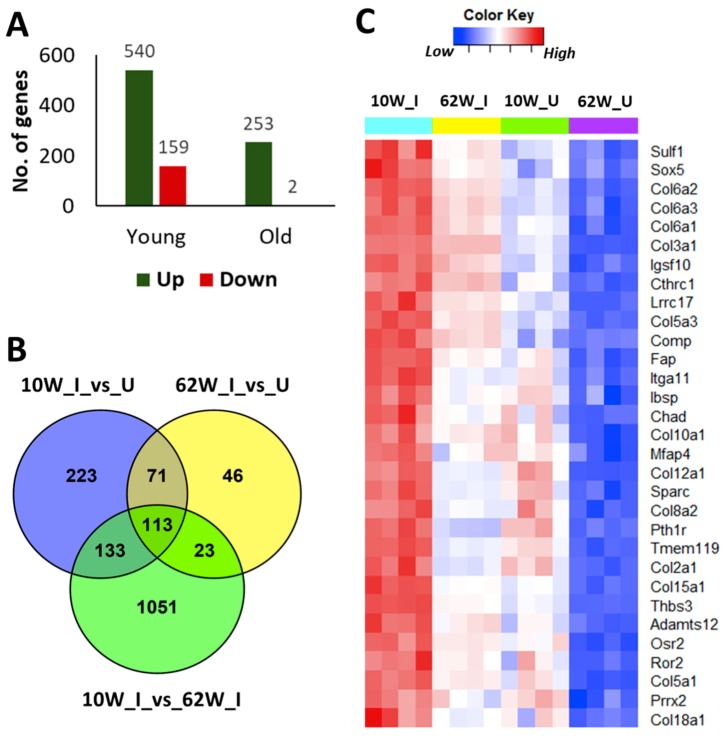
Age related differences in anterior cruciate ligament (ACL) injury-induced gene expression changes at six-week post-injury. (**A**) Number of genes differentially expressed in response to injury in 10-week-old and 62-week-old mice at six-week post-injury. (**B**) Overlap between genes up-regulated in 10-week-old and 62-week-old mice compared with respective uninjured controls and genes up-regulated in injured joints of 10-week-old compared with injured joints of 62-week-old mice. (**C**) Injury-induced regulators of cartilage and bone development/metabolism showing highest expression in injured joints of 10-week-old-mice. 10W_I: injured joints of 10-week-old; 10W_U: uninjured joints of 10-week-old; 62W_I: injured joints of 62-week-old; 62W_U: uninjured joints of 62-week-old.

To determine how aging impacts PTOA development at the molecular level, we compared injury-induced gene expression changes in both 10-week-old and 62-week-old mice at six-week post-injury. RNA-seq analysis identified 699 and 255 genes differentially expressed in injured knee joints of 10-week-old mice and 62-week-old mice, respectively, compared with respective uninjured contralateral joints (Figure 3A, Table S2). Of these genes, 184 up-regulated genes were common to both age groups (Figure 3B). These genes included 41 genes involved in extracellular matrix organization (*Htra1*, *Fn1*, *Comp*, *Tnc*, *Dcn*, *Bgn*, *Sulf1*, *Loxl1*, *Loxl2*, *Has1*, *Lum*, *Plod2*, and *Collagens*, among others), 19 genes involved in collagen metabolic process (*Collagens*, *Mmp2*, and *Mmp3*, among others), 14 genes involved in cartilage development (*Col2a1*, *Col10a1*, *Comp*, *Ror2*, *Osr2*, *Pth1r*, *Thbs3*, *Sulf1*, and *Loxl2*, among others), and 24 genes involved in bone development and metabolism (*Cthrc1*, *Ibsp*, *Sparc*, *Pth1r*, and *Enpp1*, among others). Of these common up-regulated genes, 113 genes including several regulators of cartilage and bone development/metabolism such as *Chad*, *Col10a1*, *Col2a1*, *Comp*, *Pth1r*, *Ror2*, *Ibsp*, and *Sparc* had higher expression in injured joints of 10-week-old mice compared with injured joints of 62-week-old mice, suggesting a more active cartilage and bone remodeling in young mice after injury (Figure 3B,C, Table S2). 

We also identified 356 genes up-regulated only in 10-week-old injured joints, which included several regulators of skeletal system development (*Phex*, *Sulf2*, *Fbn1*, *Fbn2*, *Sfrp1*, *Tnfrsf11b*, *Hapln1*, *Igf1*, and *Rspo2*, among others), extracellular matrix organization (*Dmp1*, *Mmp12*, *Mmp19*, *Adamts2*, *Tnc*, and *Loxl1*, among others), inflammatory response (*Cxcl10*, *C3ar1*, *C4b*, *Ccr5*, *Ccl8*, and *Il33*, among others), and Wnt signaling (*Fzd1*, *Nfatc4*, *Wnt16*, *Sfrp1*, *Sfrp4*, *Nkd2*, *Prickle2*, *Rspo2*, *Rspo1*, and *Dkk3*, among others) (Figure 3B, Table S2). Sixty-nine genes were specifically up-regulated in 62-week-old mice compared with respective uninjured controls, which included several regulators of bone development and metabolism (*Postn*, *Bmp1*, *Sfrp2*, *Gja1*, *Ptn*, *Alpl*, *Sp7*, and *Mmp14*, among others) (Figure 3B, Table S2). However, 23 of these 69 genes including *Alpl*, *Sp7*, *Bmp1*, and *Mmp14* had higher expression in both injured and uninjured joints of 10-week-old mice compared with 62-week-old mice (Table S2). Two genes (*Kcna3* and *Gm30934*) were down-regulated in 62-week-old mice, whereas 159 genes were down-regulated in 10-weekold mice in response to injury, and none of these genes overlapped with genes down-regulated in 62-week-old mice (Table S2).

**Figure 4:**
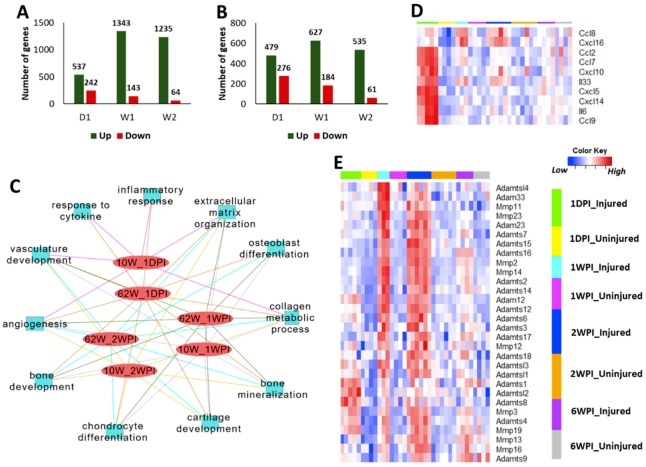
Injury-induced transcriptional changes at early post-injury timepoints. Number of genes up- and down-regulated in injured knee joints of 62-week-old (**A**) and 10-week-old (**B**) mice at one-day (1D), one-week (1W), and two weeks (2W) post-injury compared with uninjured contralateral joints. (**C**) Key biological processes associated with genes up-regulated in 62-week-old mice and 10-week-old mice at early timepoints. (**D**) Inflammatory cytokines up-regulated at one-day post-injury in 62-week-old mice. (**E**) Matrix degrading enzymes up-regulated in 62-week-old mice after injury. Majority of these had highest expression at 1–2 weeks post-injury. 10W: 10-week-old; 62W: 62-week-old; 1DPI: one-day post-injury; 1WPI: one-week post-injury; 2WPI: two weeks post-injury.

We have previously shown that, in young mice, inflammatory response genes were highly up-regulated immediately post-injury and a large number of genes associated with cartilage and bone remodeling were highly elevated at one-week and two weeks post-injury [8,10]. To identify the differences in injury-induced early molecular changes between the young and old, we profiled the knee joint transcriptome of 62-week-old mice at one-day, one-week, and two weeks post-injury. Our analysis identified 779, 1486, and 1299 genes differentially expressed in injured knee joints of old mice at one-day, one-week, and two-weeks post injury, respectively, compared with respective uninjured contralateral joints (Figure 4A, Table S2). In 10-week-old mice, 755, 811, and 596 genes were differentially expressed at one-day, one-week, and two weeks post injury, respectively (Figure 4B, Table S2). We also observed a huge overlap between genes up-regulated in both age groups at early post-injury timepoints (Table 1). This included 48 genes up-regulated in both age groups at all post-injury timepoints examined in this study (Table 2).

Injury-induced genes in both young and old mice showed enrichment for similar biological processes at early post-injury timepoints (Figure 4C). Similar to what we have previously shown for young mice [10], 62-week-old mice also displayed an up-regulation of inflammatory response related genes at one-day post-injury (Figure 4C,D, Table S2). These genes included several cytokines that also showed an increase in expression with age such as *Ccl8*, *Cxcl5*, and *Il33* (Table S1). As in the case of young mice [10], a large number of genes involved in extracellular matrix organization and cartilage/bone metabolism including core matrix proteins such as collagens, *Fn1*, *Dcn*, *Eln*, *Fbn1*, *Fbn2*, *Bgn*, *Acan*, *Cthrc1*, *Postn*, *Prelp*, *Prg4*, and *Vcan* and matrix degrading enzymes such as *Mmp2*, *Mmp3*, *Adamts2*, and *Adamts3* showed an increased expression in the injured joints of 62-week-old mice compared with uninjured controls (Figure 4E, Figure S2, Table S2). The majority of these genes had the highest expression at 1–2 weeks post-injury, indicating a more active tissue remodeling at this timepoint (Figure 4E, Figure S2). We also observed that a number of collagen processing enzymes such as *Lox*, *Loxl2*, *Loxl3*, and *Plod2* were activated by injury in both age groups (Figure S3A). Using single-cell RNAseq (scRNA-seq) data from adult mouse knee joint cartilage, we also determined that mature chondrocytes robustly express *Loxl2*, *Loxl3*, and *Plod2*, whereas *Lox* expression was more restricted to immature chondrocytes/mesenchymal progenitors (Figure S3B). 

**Figure 5:**
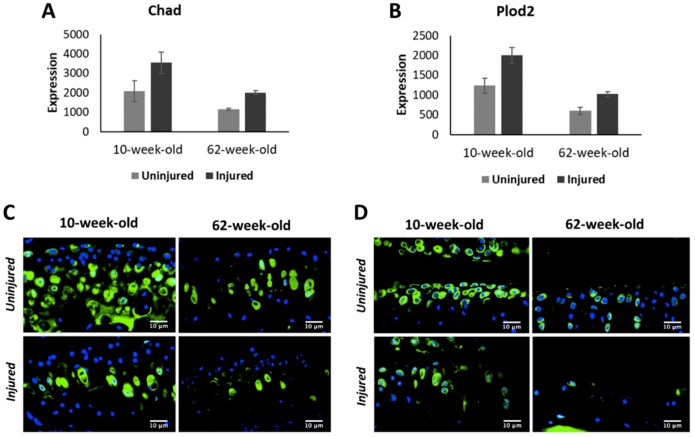
*Chad* (**A**) and *Plod2* (**B**) gene expression at six-week post injury in 10-week-old and 62-week-old mice. Chad (**C**) and Plod2 (**D**) protein expression in 10-week-old and 62-week-old mice Green: protein staining. Blue: DAPI staining marking the nucleus. (40× magnification).

Although injury activated a large number of genes involved in cartilage anabolism in both age groups, histological analysis (Figure 2) suggested that this was not sufficient to prevent joint degeneration. To further investigate this, we performed immunohistochemistry analysis of two injury-induced genes: chondroadherin (*Chad*), a leucine rich repeat extracellular matrix protein that is synthesized by chondrocytes and reported to promote their attachment [12]; and *Plod2*, an enzyme involved in collagen synthesis [13]. Consistent with gene expression data (Table S1), immunohistochemistry showed lower Chad and Plod2 protein expression in the articular cartilage of old mice compared with young mice, confirming a decrease in expression with age (Figure 5C,D). However, in contrast to an increase in the transcript levels observed after injury (Table S2), injured joints of both age groups had significantly lower Chad and Plod2 protein expression compared with respective uninjured controls, suggesting that the increase in gene expression in response to injury failed to translate into increased protein expression. Alternatively, other cells in the joint may have up-regulated the transcript levels of these genes, in which case the non-cartilage derived RNA would account for higher transcript levels in the injured joints.

**Figure 6:**
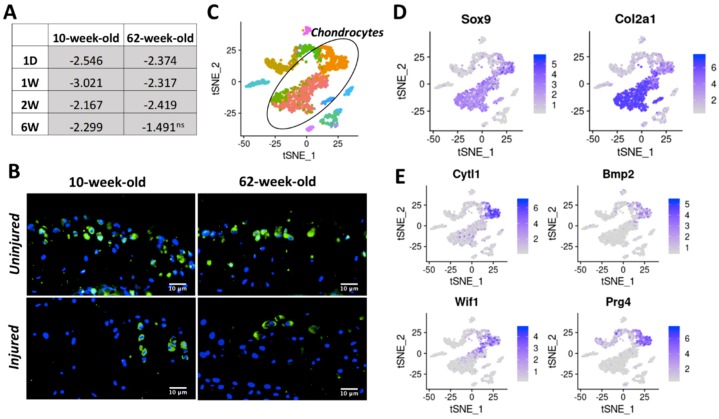
(**A**) Table showing *Cytl1* fold down-regulation in injured joints compared with uninjured controls at various post-injury timepoints, in both 10-week-old and 62-week-old mice. (**B**) Cytl1 protein expression at six-week post injury in 10-week-old and 62-week-old mice. Green: protein staining. Blue: DAPI staining marking the nucleus. (40× magnification) (**C**) tSNE plot of mouse cartilage cells identified using scRNA-seq. Each color represents a distinct cell type/subtype. Chondrocyte subtypes identified in mouse cartilage based on the expression of chondrocyte markers *Col2a1*, *Acan*, and *Sox9* are shown in black oval. (**D**) Expression of chondrocyte markers *Sox9* and *Col2a1* in scRNA-seq data. (**E**) *Cytl1* expression is restricted to a chondrocyte subtype that also express high levels of *Bmp2*, *Wif1*, and *Prg4* at high levels. Ns: not significant.

The number of genes down-regulated in response to injury was much lower than the number of up-regulated genes in both age groups, at all timepoints examined (Figure 4A,B). At one-day post-injury, 68 genes were down-regulated in both 10-week-old and 62-week-old mice including several genes involved glucose catabolic process in such as *Pfkfb1*, *Pgam2*, and *Eno3* (Table S2). Thirty-seven genes including several regulators of muscle structure and function such as *Myh1*, *Myh2*, *Myl2*, *Myl3*, *Actn2*, and *Ankrd2* were down-regulated in both age groups at one-week post-injury (Table S2). Cytokine-like 1 (*Cytl1*) [14,15], a gene potentially involved in chondrogenesis and cartilage development, was down-regulated in 10-week-old mice at all post-injury timepoints and in 62-weeks old mice at one-day, one-week, and two weeks post-injury (Figure 6A), and this trend was consistent when protein levels were analyzed by immunohistochemistry (Figure 6B), suggesting that *Cytl1* correlates with PTOA severity. scRNA-seq analysis of adult mouse knee joint cartilage showed that *Cytl1* was robustly expressed by a specific chondrocyte subpopulation, which also expressed high levels of *Bmp2*, *Wif1*, and *Prg4*, genes that play a role in chondrocyte differentiation and maintenance [16,17,18] (Figure 6C–E), indicating a spatially restricted expression pattern for this gene in the cartilage and a potential role in the development and maintenance of the articular cartilage. 

## 3. Discussion

Aging and injury are two key risk factors for OA. Several studies have investigated how aging and injury independently contribute to OA pathogenesis; however, very limited data are available on how aging and injury interact to influence OA progression [4]. In this study, we investigated the differences in molecular responses of young and old mice to knee joint injury using RNA-seq. Our study identified several age-related structural and molecular changes in mouse knee joint during PTOA development and progression.

Histological analysis showed that both 10-week-old and 62-week-old mice exhibited significant joint degeneration by six-week post-injury. Consistent with previous reports [4], older mice had more severe articular cartilage degeneration compared with young mice. Both age groups had deficits in epiphyseal trabecular bone in the injured joint and exhibited considerable osteophyte formation, which was more severe in older animals. Old mice also showed lower trabecular bone volume fraction (BV/TV) compared with the young mice, suggesting an age-dependent bone loss. Our previous study showed peak trabecular bone changes at approximately two weeks post injury, while mineralized osteophytes were not observable by μCT until about four weeks [19]. In this study, we only examined six-week post-injury joints using μCT and histology, and the joints were severely damaged in both age groups by six-week post-injury. Examining an earlier time point (e.g., 2–4 weeks post-injury) would potentially allow us to more clearly see differences in PTOA progression between old and young mice and determine whether aging accelerates PTOA. 

At a molecular level, older mice had significantly higher expression of inflammatory signaling genes including *Ccl3*, *Ccl4*, *Ccl5*, *Ccl8*, *Cxcl5*, *Cxcl9*, *Cxcl13*, *Il6*, and *Il33* compared with young mice and the expression of many of these inflammatory cytokines increased with age, with 95-week-old mice showing the highest expression (Figure 1C). A study comparing gene expression in joint tissues from 12-week-old mice and 12-month-old mice has shown that the expressions of *Il6*, *Il33*, *Cxcl9*, *Cxcl13*, *Ccl8*, and *Ccl5*, were significantly up-regulated in the old mice compared with the young [4], which is consistent with our observation. We also found that knee joint injury further activated the expression of many of these genes in both young and old mice at one-day post-injury, and the majority of these genes reverted to the uninjured control level by 1–2 weeks post-injury in both age groups. It has been suggested that inflammation plays a key role in the pathogenesis of osteoarthritis; inflammatory mediators may promote cartilage degradation either directly or indirectly through the induction of proteolytic enzymes [20,21]. Low innate production of interleukin (IL)-6 has been shown to be associated with the absence of osteoarthritis in old age [22]. It has also been shown that STR/ort, a mouse strain with high susceptibility to OA, expresses high levels of inflammatory markers, whereas MRL/MpJ, a mouse strain resistant to PTOA, had low expression values for these genes [10,23,24]. These data together suggest that an increase in the levels of inflammatory mediators with age might have played a role in the enhanced joint degeneration observed in older mice.

Consistent with previous studies demonstrating an age-related decline in chondrocyte anabolic responses [4,25], we observed a significant reduction in the expression of genes involved in cartilage development and metabolism including *Sox9* [26], *Col2a1*, *Col9a1-a3* [27], *Acan*, *Comp*, *Perlecan (Hspg2)* [28], and *Hapln1* [29] with age (Table S1). *Bmp7*, a key regulator cartilage homeostasis and cartilage repair [30], also showed reduced expression in old animals. Chondroadherin (Chad), a cartilage matrix protein thought to mediate chondrocyte adhesion, also displayed a down-regulation with age [12]. However, knee joint injury activated the expression of many of these cartilage anabolic genes in both 10-week-old and 62-week-old mice (Table S2). These observations are consistent with previous data showing up-regulation of these genes in human OA cartilage compared with normal cartilage [31]. We also found that injured joints of 10-week-old mice had higher expression of these genes compared with injured joints of 62-week-old mice, suggesting that young mice are more actively trying to repair the cartilage after injury (Figure 3C). However, immunohistochemical analysis of Chad showed a significant reduction in protein expression in the injured joints of both 10-week-old and 62-week-old mice at six-week post-injury. This suggests that increased expression of cartilage anabolic genes after injury was not sufficient to prevent cartilage degeneration. 

Cartilage intermediate layer protein 2 (CILP-2) is a protein that is localized in the deeper intermediate zone of the articular cartilage extracellular matrix and was down-regulated in mouse OA cartilage [32]. Serum levels of CILP-2 appear to be associated with loss of cartilage thickness in certain individuals with increased risk of developing knee osteoarthritis [33]. We found that *Cilp2* showed an age-dependent decrease in expression (Table S1). Our data also showed down-regulation of *Cilp2* immediately post-injury in both 10-week-old and 62-week-old mice; however, it was up-regulated at later timepoints in both age groups (Table S2). Several genes involved in matrix degradation including MMPs (*Mmp2*, *-3*, *-12*, *-14*, *-19*), ADAMTS (*Adamts4*, *-6*, *-12*, *-15*, *-16*) and HtrA serine peptidase 1 and 3 were also up-regulated in both young and old mice after injury and may play a role in tissue remodeling after joint injury.

Expression of collagen crosslinking enzymes lysyl oxidases (*Lox*, *Loxl2*, and *loxl3*) also decreased with age (Figure S3C). Lysyl oxidases play a key role in physiological and pathological remodeling of extracellular matrix and it has been shown that systemic LOXL2 adenovirus or LOXL2 genetic overexpression in mice can protect against OA [34]. Procollagen-lysine,2-oxoglutarate 5-dioxygenase 2 (*Plod2*), another collagen processing enzyme, also had significantly lower expression in old mice compared with young. Mutations in *PLOD2* cause Bruck syndrome (BS), a rare congenital connective tissue disorder characterized by a combination of joint contractures with various skeletal anomalies [35]. It has been suggested that PLOD2 plays a role in synovial fibrosis, a major contributor to joint stiffness in OA [36]. scRNA-seq analysis of adult mouse knee joints cartilage indicated that chondrocytes robustly express this gene (Figure S3B), suggesting that Plod2 plays a role in maintaining cartilage homeostasis. We observed a significant up-regulation of transcripts encoding these collagen processing enzymes after injury in both age groups at various post injury timepoints, possibly as a mechanism to prevent cartilage damage (Table S2). However, immunohistochemistry analysis of Plod2 showed a decrease in protein expression in injured joint at six-week post-injury, which was consistent with what we observed for cartilage matrix protein Chad (Figure 5C,D). We only examined the cartilage for protein expression, and other cells in the joint such as osteoblasts, fibroblasts, or immune cells may have contributed to the up-regulation of the transcript levels of these genes, in which case the non-chondrocyte derived RNA would account for higher transcript levels in the injured joints. Differences in the rates of RNA and protein metabolism could also have contributed to this inconsistency. Further studies are required to understand how protein metabolism is regulated after injury and how this plays a role in cartilage degeneration.

Very few genes were down-regulated in both age groups after injury. Cytokine-like 1 (CYTL1) is a cytokine that has been shown to promote chondrogenic differentiation of mesenchymal stem cells [14], and *Cytl1* knock-out mice were more sensitive to osteoarthritic (OA) cartilage destruction than wildtype mice [15]. Cytokine-like 1 (CYTL1) was down-regulated in both young and old mice after injury (Table S2). Cytl1 also showed a reduced expression in 62-week-old mice compared with 10-week-old mice. Furthermore, we found that in adult mice *Cytl1* expression is restricted to a subset of chondrocytes, which also express high levels of *Bmp2*, a key regulator of chondrogenic differentiation [16]; Wnt inhibitor *Wif1*; and lubricin (*Prg4*), a gene involved in boundary lubrication [37] (Figure 6C–E). Wif1 is expressed at cartilage-mesenchyme interfaces and neutralizes Wnt3a-mediated inhibition of chondrogenesis [17]. It has been shown that Prg4-expressing cells located at the embryonic joint surface serve as a progenitor population for all deeper layers of the mature articular cartilage [38]. It has also been shown that Prg4 plays an important anti-inflammatory role in regulating synoviocyte proliferation in OA and reduces basal and IL-1β-stimulated expression of matrix degrading enzymes [18]. Co-localization of Cytl1 with these genes indicate that Cytl1 plays a role in chondrocyte differentiation and maintenance. Consistent with our RNA-seq data, immunohistochemical analysis showed that Cytl1 expression is reduced after injury in both age groups (Figure 6A,B). Our data suggest that age- or injury-induced decrease of Cytl1 could negatively affect cartilage homeostasis and contribute to joint degeneration.

Our study shows clear age-related structural and transcriptional differences in the murine knee joints after injury. One major limitation of this study is the use of mouse models instead of human subjects. The murine models may not fully recapitulate the changes seen in the human joints as a result of age or injury. Several studies have used human biopsy samples to investigate OA pathogenesis, but there are limitations in terms of the types of studies that can be conducted using human subjects as it is difficult to obtain biopsy samples from healthy and diseased human knee joints at multiple timepoints. However, murine models allow us to overcome some of the limitations of human data, by allowing us to conduct longitudinal studies and gain insights into PTOA development and progression. Another limitation of our study is that injury-induced transcriptional changes were identified relative to uninjured contralateral joint, which may have caused us to underestimate injury-induced systemic changes that may affect both joints. Our previous studies have shown that the expression of several inflammatory cytokines was up-regulated in both injured and uninjured contralateral joints in response to injury, although the level of activation in the contralateral was significantly lower than injured joints. Using contralateral joints as controls may not accurately capture such injury-induced systemic changes. Also, transcriptome data were obtained from whole joints, which makes it difficult to tease out the cell type-specific gene expressions changes. To overcome this challenge, we have examined tissue specific expression of selected proteins using other techniques such as immunohistochemistry and scRNA-seq. Nevertheless, this study provides novel insights into genes and molecular pathways involved in the PTOA development in young and old. Our data suggest that increased inflammation and reduced cartilage anabolism as a result of aging may contribute to a severe PTOA phenotype in old individuals. Keeping inflammation under control after joint injury may be beneficial in preventing or at least slowing down cartilage damage. This study also identified several potential therapeutic targets for PTOA including collagen metabolism enzymes such as lysyl oxidases and Plod2 and proteins such as Cytl1. In summary, this study highlights several new genes and molecular pathways that play a role in PTOA pathogenesis in young and old mice, and the data presented herein could help facilitate future research, which could aid the development of novel therapeutic approaches for PTOA.

## 4. Materials and Methods

### 4.1. Tibial Compression Overload Injury 

Right knee joints of 10-week-old and 62-week-old C57BL/6J mice (Jackson Laboratory, Bar Harbor, ME, USA; Stock No: 000664) received a single non-invasive tibial compression overload at 1 mm/s displacement rate until ACL rupture using an electromagnetic material testing system (ElectroForce 3200, TA Instruments, New Castle, DE, USA), as previously described [8,10,19]. Buprenorphine was administered immediately post-injury (0.01 mg/kg) for pain relief. All animal experimental procedures were completed in accordance with the Institutional Animal Care and Use Committee (IACUC) guidance at Lawrence Livermore National Laboratory and the University of California, Davis in AAALAC-accredited facilities under protocol 168 approved in February 2019.

### 4.2. Histological Assessment of Disease Severity 

Injured and uninjured (contralateral) joints were collected at six-week post-injury and processed as previously described for histology [10]. Briefly, dissected joints were fixed in 4% paraformaldehyde, decalcified using 0.5 M EDTA, processed, and embedded intact into paraffin. The joints were sectioned in the sagittal plane and serial medial sections that included the femoral condyles, menisci, and tibial plateaus were cut at 4 μm; stained on glass slides using 0.1% Safranin-O (0.1%, Sigma, St. Louis, MO, USA; S8884) and 0.05% Fast Green (0.05%, Sigma, St. Louis, MO, USA; F7252) using standard procedures (IHC World, Woodstock, MD, USA); and imaged using a Leica DM5000 microscope. Blinded slides were evaluated by seven scientists (six with and one without expertise in OA) utilizing modified (sagittal) Osteoarthritis Research Society International (OARSI) scoring parameters because of the severity of the phenotype six weeks following TC injury. Modified scoring scores (0) for intact cartilage staining with strong red staining on the femoral condyle and tibia, (1) for minor fibrillation without cartilage loss, (2) for clefts below the superficial zone, (3) for cartilage thinning on the femoral condyle and tibia, (4) for lack of staining on the femoral condyle and tibia, (5) for staining present on 90% of the entire femoral condyle with tibial resorption, (6) for staining present on over 80% of the femoral condyle with tibial resorption, (7) for staining present on 75% of the femoral condyle with tibial resorption, (8) for staining present on over 50% of the femora condyle with tibial resorption, (9), for staining present in 25% of the femoral condyle with tibial resorption, and (10) for staining present in less than 10% of the femoral condyle with tibial resorption.

### 4.3. Micro-Computed Tomography (μCT)

Whole knee joints from both young and old mice (*n* ≥ 5 per group) were scanned using μCT (SCANCO μCT 35, Brüttisellen, Switzerland) at six-week post injury as previously described [10], according to the rodent bone structure analysis guidelines (X-ray tube potential = 55 kVp, intensity = 114 μA, 10 μm isotropic nominal voxel size, integration time = 900 ms). Trabecular bone in the distal femoral epiphysis was analyzed by manually drawing contours on 2D transverse slides. The distal femoral epiphysis was designated as the region of trabecular bone enclosed by the growth plate and subchondral cortical bone plate. Epiphyseal trabecular bone volume fraction was determined by quantifying trabecular bone volume per total volume (BV/TV). Mineralized osteophyte volume in injured and contralateral joints was quantified by drawing contours around all heterotropic mineralized tissue attached to the distal femur and proximal tibia as well as the whole fabellae, menisci, and patella. Total mineralized osteophyte volume was then determined as the volumetric difference in mineralized tissue between injured and uninjured joints. Statistical analysis was performed using a paired *t*-test to compare injured and contralateral knees.

### 4.4. Immunohistochemistry (IHC)

Sagittal six-micrometer sections from injured and uninjured samples from both age groups of C57Bl/6J mice were used for IHC. Unitrieve was used as an antigen retrieval method for 30 min at 65 °C. Primary antibodies: CYTL1 (Proteintech, Rosemont, IL, USA; 15856-1-AP(1:75)), PLOD2 (Invitrogen, Carlsbad, CA, USA; PA5-69194(1:100)), and CHAD (Invitrogen, Carlsbad, CA, USA; PA5-53761(1:300)) were used and incubated overnight at room temperature in a dark, humid chamber. Negative control slides were incubated with secondary antibody-only. Stained slides were mounted with Prolong Gold with DAPI (Molecular Probes, Eugene, OR, USA). Slides were imaged using a Leica DM5000 microscope. ImagePro Plus V7.0 Software and a QIClick CCD camera (QImaging, Surrey, BC, Canada) were used for imaging and photo editing.

### 4.5. Bulk RNA Sequencing and Data Analysis

The knee joint RNA from 10-week-old, 62-week-old, and 95-week-old mice was isolated as described before [10]. Briefly, injured and contralateral joints (*n* = 3–6 per group) were dissected, chopped, and homogenized in Qiazol (79306, Qiagen, Valencia, CA, USA). Total RNA was purified using RNeasy Mini Kit (QIAGEN Inc., Germantown, MD, USA) according to the manufacturer’s protocol and the RNA integrity was assessed using a bioanalyzer (Agilent Technologies, Santa Clara, CA, USA). Poly(A)+-enriched cDNA libraries were generated using the Illumina TruSeq RNA Library Prep kit v2 (Illumina Inc., Hayward, CA, USA). The sequencing was performed using an Illumina (Illumina Inc., Hayward, CA, USA) NextSeq 500 instrument to generate 75 bp single-end reads. The quality of sequencing data was checked using FastQC (version 0.11.5) software [http://www.bioinformatics.bbsrc.ac.uk/projects/fastqc]. Sequence reads were mapped to the mouse reference genome (mm10) using STAR (version 2.6) [39]. A matrix of raw counts per gene was generated using “featureCounts” from Rsubread package (version 1.30.5) [40]. RUVseq was used to determine factors of unwanted variation [41]. Differentially expressed genes were identified using edgeR, adjusting for factors of unwanted variation [42]. Genes with fold changes >1.5 and false discovery rate (FDR) adjusted *p*-value less than 0.05 were considered as significantly differentially expressed. Heatmaps were generated using heatmap.2 function in R package ‘gplots’.

### 4.6. Single Cell RNA-Seq (scRN-Aseq) and Data Analysis

Ten-week-old C57Bl/6J mice were euthanized, and right and left hindlimbs were collected by removing the leg at the hip joint and storing on ice in Dulbecco’s Modified Eagle Medium Nutrient Mixture F-12 (DMEM/F-12) (Thermo Fisher Scientific, Waltham, MA, USA). The articular cartilage of the knee joint was isolated from the femur and tibia by cutting 0.5–1 mm of tissue from the end of both long bones. Cartilage tissue was digested to a single cell suspension in 1 mL of 0.2% Collagenase 2 solution (Thermo Fisher Scientific, Waltham, MA, USA) while shaking at 37 °C for 2 h. Fractions were collected at 30 min intervals, filtered through a 70 μm Nylon cell strainer, and then resuspended in DMEM/F12 with 10% fetal bovine serum (FBS). Remaining undigested cartilage tissue was further incubated in 1 mL of fresh Collagenase 2 digestion media. 

Following digestion, cell suspensions were pelleted via centrifugation for 10 min at 500 G, then resuspended in ACK lysis buffer (Thermo Fisher Scientific, Waltham, MA, USA), and incubated for 10 min on ice in order to remove red blood cells. Cell suspensions were next resuspended in 100 uL of PBS + 10% FBS. CD45+ and Ter119+ cell depletion was accomplished using CD45 and Ter119 MACSmicrobeads (Miltenti Biotec, Sunnyvale, CA, USA) and running cell samples on an LS MACScolumns (Miltenti Biotec, Sunnyvale, CA, USA) following the manufacturer’s instructions. Final cell counts were performed using a hemocytometer and cells were resuspended in PBS + 0.04% nonacetylated BSA before introduction into a Chromium Controller (10x Genomics, Pleasanton, CA, USA). Library preparation was performed using Chromium Single Cell 3′ GEM, Library & Gel Bead Kit v3 (10x Genomics, Pleasanton, CA, USA; Catalog no. 1000075) following the manufacturer’s protocol and sequenced using Illumina NextSeq 500. Sequencing data were demultiplexed, quality controlled, and aligned to the mouse genome (mm10) using Cell Ranger (10x Genomics, Pleasanton, CA, USA). Data analysis was performed using Seurat (version 3.1.1) [43].

### 4.7. Functional Annotation

Gene ontology analysis was performed using ToppGene and ToppCluster [44] and enriched gene ontology terms and pathways (*p*-value < 0.01) were identified. Cytoscape (version 3.6.1) was used for ontology and pathway visualization [45].

## Figures and Tables

**Table 1:** Table showing up-regulated genes shared between 10-week-old and 62-week-old mice at various post-injury timepoints.

Time Post-Injury		62-Week-Old			
		1 Day (Total: 537)	1 Week (Total: 1343)	2 Weeks (Total: 1235)	6 Weeks (Total: 253)
10-week-old	1 Day (Total: 479)	246	288	256	78
	1 Week (Total: 627)	227	536	534	179
	2 Weeks (Total: 535)	196	465	462	177
	6 Weeks (Total: 540)	170	427	430	184

**Table 2:** Genes up-regulated in injured joints of both young and old mice at all timepoints examined. Fold change (log2 scale) values are shown in the table (false discovery rate (FDR) < 0.05 for all genes).

Age	Young				Old			
Gene	1 Day	1 Week	2 Weeks	6 Weeks	1 Day	1 Week	2 Weeks	6 Weeks
*Adamts12*	0.649	1.324	0.917	0.783	1.225	2.363	1.898	0.804
*AW551984*	1.397	1.863	1.662	1.048	1.479	2.865	2.787	1.106
*Bmper*	1.402	1.245	1.039	1.023	1.436	1.447	1.443	0.813
*Col14a1*	1.112	2.110	1.552	1.424	0.730	2.939	2.508	1.016
*Col18a1*	0.741	1.263	1.124	0.606	1.003	2.268	1.758	0.689
*Col3a1*	1.543	2.075	1.683	1.594	2.395	4.267	3.878	2.270
*Col5a1*	0.916	1.441	0.995	0.781	1.053	3.080	2.482	1.078
*Col5a2*	0.725	1.157	0.793	1.036	1.304	3.118	2.815	1.381
*Col5a3*	0.670	1.119	0.735	1.080	0.690	2.137	1.783	1.007
*Col6a1*	0.752	1.674	1.282	0.992	0.934	3.155	2.603	1.256
*Col6a2*	0.752	1.688	1.287	1.162	0.993	3.170	2.639	1.156
*Col6a3*	0.930	1.807	1.156	1.292	1.436	3.188	2.564	1.270
*Col8a1*	0.609	0.748	0.615	0.590	0.965	1.681	1.537	0.701
*Crabp2*	1.600	2.440	2.452	1.956	1.842	3.802	4.167	1.867
*Cthrc1*	1.686	2.050	1.281	1.488	1.765	4.493	3.955	1.914
*Dab2*	0.994	1.056	0.888	0.606	1.200	1.697	1.668	0.772
*Dsel*	0.692	1.267	0.736	0.919	0.686	1.764	1.700	1.005
*Enpp1*	0.685	1.408	0.789	1.393	0.860	1.914	2.009	1.265
*Fcrls*	1.990	1.288	1.208	0.895	2.233	2.501	2.223	1.005
*Fndc1*	0.825	1.977	1.474	1.413	0.668	2.795	2.227	1.023
*Fstl1*	1.196	1.500	1.217	0.895	1.278	2.371	2.128	0.745
*Has1*	1.367	1.921	1-.955	3.181	3.310	2.894	2.648	1.590
*Hhipl1*	1.551	1.644	1.067	1.431	1.357	1.896	1.780	1.386
*Igsf10*	0.990	1.772	1.072	1.405	0.750	2.965	2.662	1.479
*Kcnj15*	1.108	2.295	1.724	2.637	1.566	2.779	2.562	1.192
*Lrrc17*	2.079	1.925	1.112	0.871	1.316	2.565	2.359	0.912
*Mfap5*	1.189	1.732	1.322	1.094	1.214	2.235	2.038	0.893
*Mmp3*	1.648	1.988	2.032	1.094	3.513	2.440	3.143	2.060
*Mrgprf*	1.071	1.967	1.493	1.309	0.848	2.582	2.138	0.988
*Nox4*	1.343	1.028	1.303	1.185	1.240	1.604	1.489	1.066
*P4ha3*	2.305	2.528	1.917	1.187	2.206	4.191	3.840	1.621
*Plce1*	0.648	0.863	0.672	0.668	0.657	1.160	1.099	0.704
*Prg4*	0.733	1.228	1.273	1.817	1.618	1.526	1.471	1.620
*Prrx2*	1.492	1.385	1.176	0.838	1.773	3.217	2.546	1.032
*Ptgfrn*	0.726	1.164	0.802	0.754	1.131	2.010	1.697	0.775
*Slc16a2*	1.413	1.655	0.950	0.792	1.360	1.783	1.654	0.782
*Slc1a4*	0.984	1.629	1.206	1.143	1.566	2.347	1.991	1.319
*Slc41a2*	0.727	0.814	0.619	0.621	1.026	1.096	1.438	0.711
*Srpx2*	0.842	1.219	0.867	1.054	1.207	2.155	2.032	0.839
*Sulf1*	0.896	1.245	0.911	1.029	1.333	1.743	1.713	0.903
*Thbs2*	0.958	1.614	0.831	0.733	0.684	2.893	2.294	1.086
*Thbs3*	0.643	1.773	1.330	1.429	1.115	3.018	2.660	1.214
*Thbs4*	0.832	0.925	0.974	0.695	1.349	1.717	1.546	1.052
*Timp1*	2.037	1.193	1.059	1.132	4.129	2.886	2.697	1.377
*Tmem45a*	0.606	1.129	0.788	0.940	1.361	2.375	2.159	0.990
*Tnfaip6*	1.941	1.804	1.345	2.636	3.897	3.911	3.240	1.722
*Tnn*	1.117	1.722	1.019	1.056	1.098	4.072	3.469	1.830

## Supplementary Materials

Supplementary materials can be found at https://www.mdpi.com/1422-0067/21/1/364/s1.

## Abbreviations

ACL	Anterior cruciate ligament
OA	Osteoarthritis
PTOA	Post-traumatic osteoarthritis
scRNA-seq	Single-cell RNA sequencing
TC	Tibial compression
μCT	Microcomputed tomography
