# Supplementary material for: Global Gene Expression Analysis Identifies Age-Related Differences in Knee Joint Transcriptome during the Development of Post-Traumatic Osteoarthritis in Mice
Source: Int J Mol Sci. 2020 Jan 6;21(1):364. doi: 10.3390/ijms21010364 (PMC6982134; doi:10.3390/ijms21010364)

Figure S1. Key genes differentially expressed between young and old mice. A) arthritis-associated genes up-regulated in old mice compared to young mice. Chondrocyte differentiation (B), bone development (C) and osteoblast differentiation associated genes upregulated in young mice compared to old mice. 10W: 10-week-old; 62W: 62-week-old; 95W: 95-week-old.

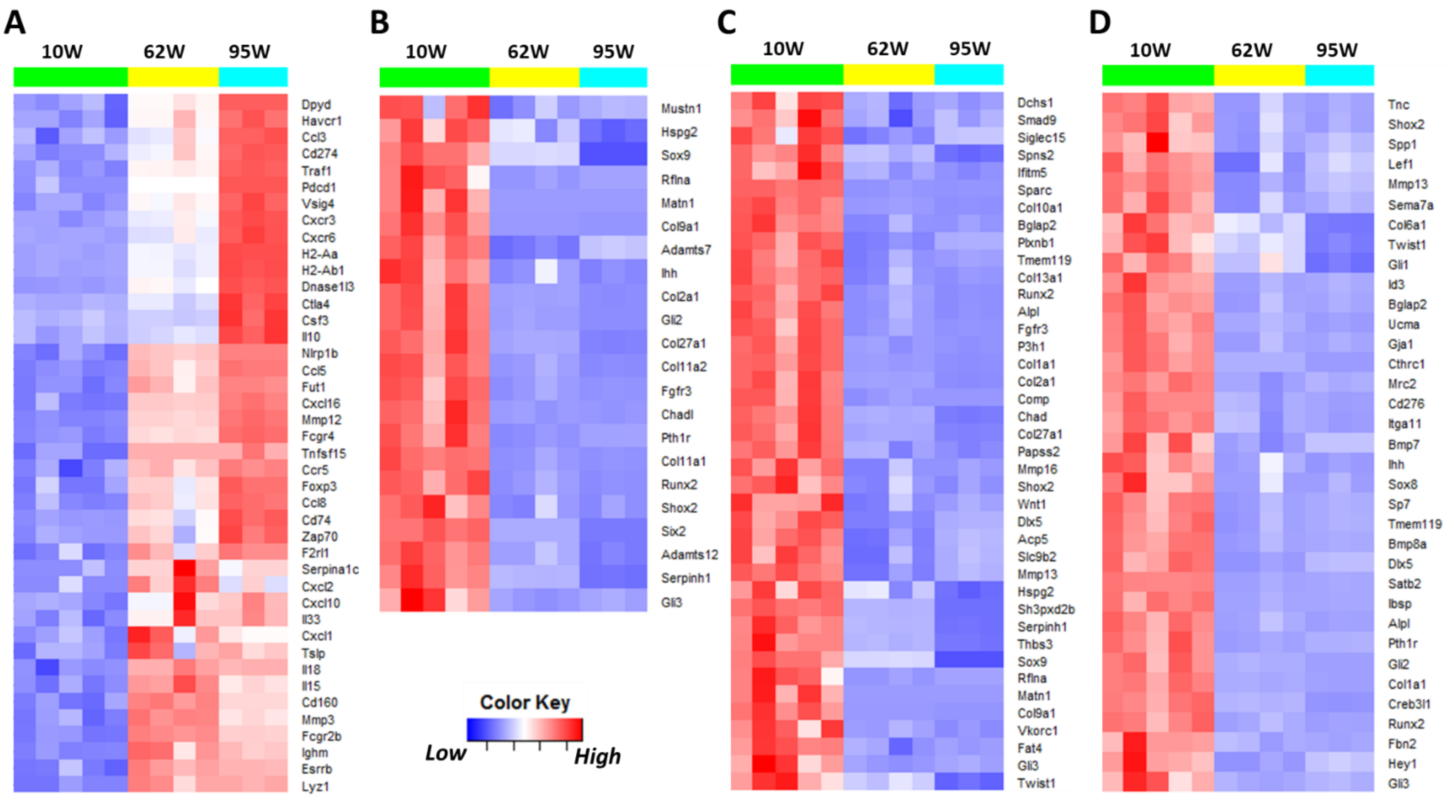

Figure S2. Extracellular matrix proteins up-regulated after injury in 62-week-old mice. Majority of these genes had highest expression at 1-2 weeks post-injury. 10W: 10-week-old; 62W: 62-week-old; 1DPI : 1-day post-injury; 1WPI: 1-week post-injury; 2WPI: 2-weeks post-injury; 6WPI: 6-weeks post-injury.

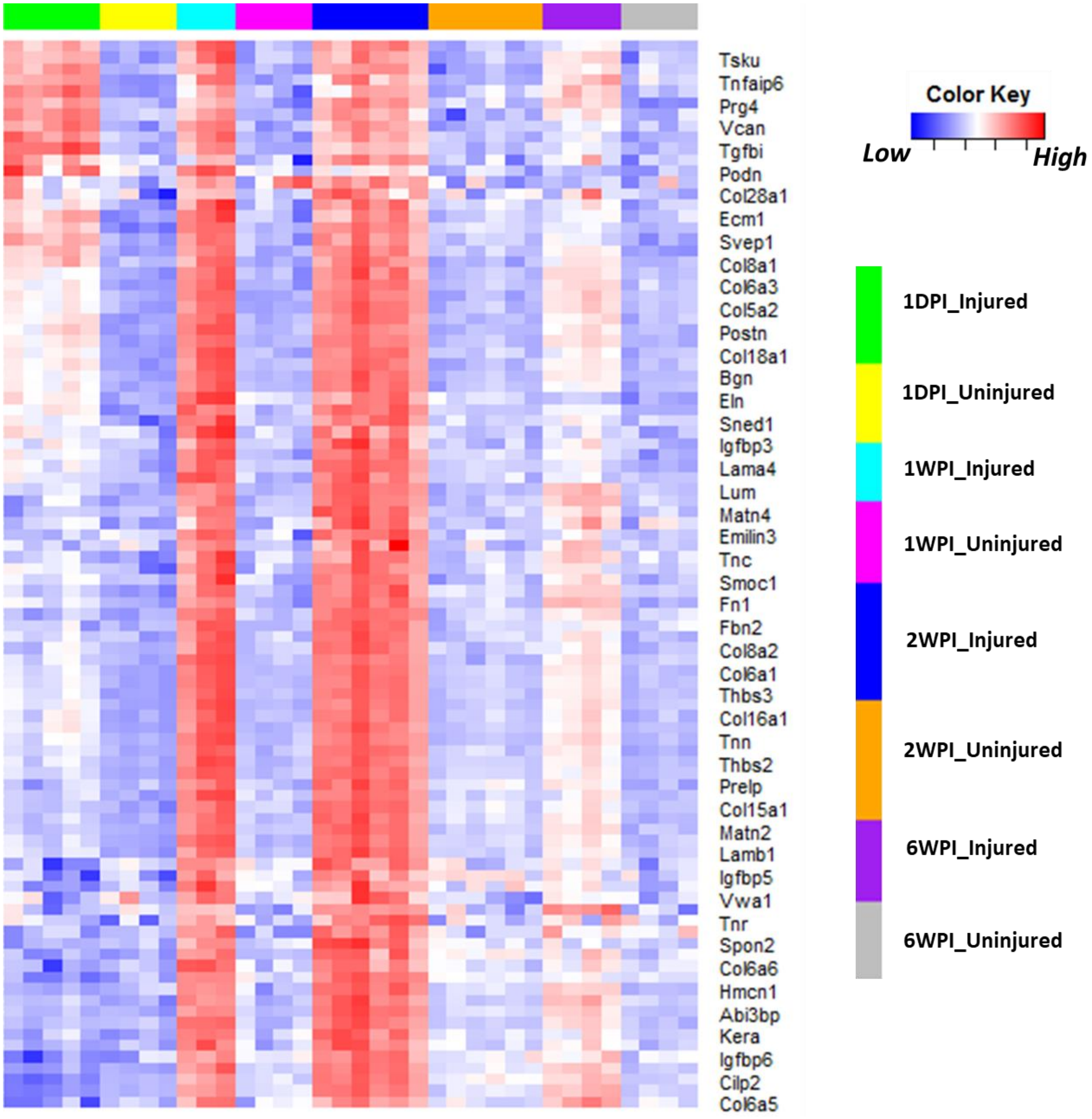

Figure S3. Collagen processing enzymes up-regulated after injury in both 10-week-old mice and 62-week-old mice. A) Fold upregulation of collagen processing enzymes in injured joints of 10-week-old (young) and 62-week-old(old) mice relative to respective uninjured controls. B) scRNA-seq plots showing the expression of chondrocyte markers *Sox9*, *Col2a1* and *Acan*, mesenchymal stem cell markers *Thy1* and *Pdgfra* and collagen processing enzymes in knee joint cartilage from 10-week-old mice. Chondrocytes robustly expressed *Plod2*, *Loxl2* and *Loxl3* whereas *Lox* was mostly expressed by mesenchymal progenitors/chondrocyte progenitors. D) Graphs showing aging-induced down-regulation of collagen processing enzymes.

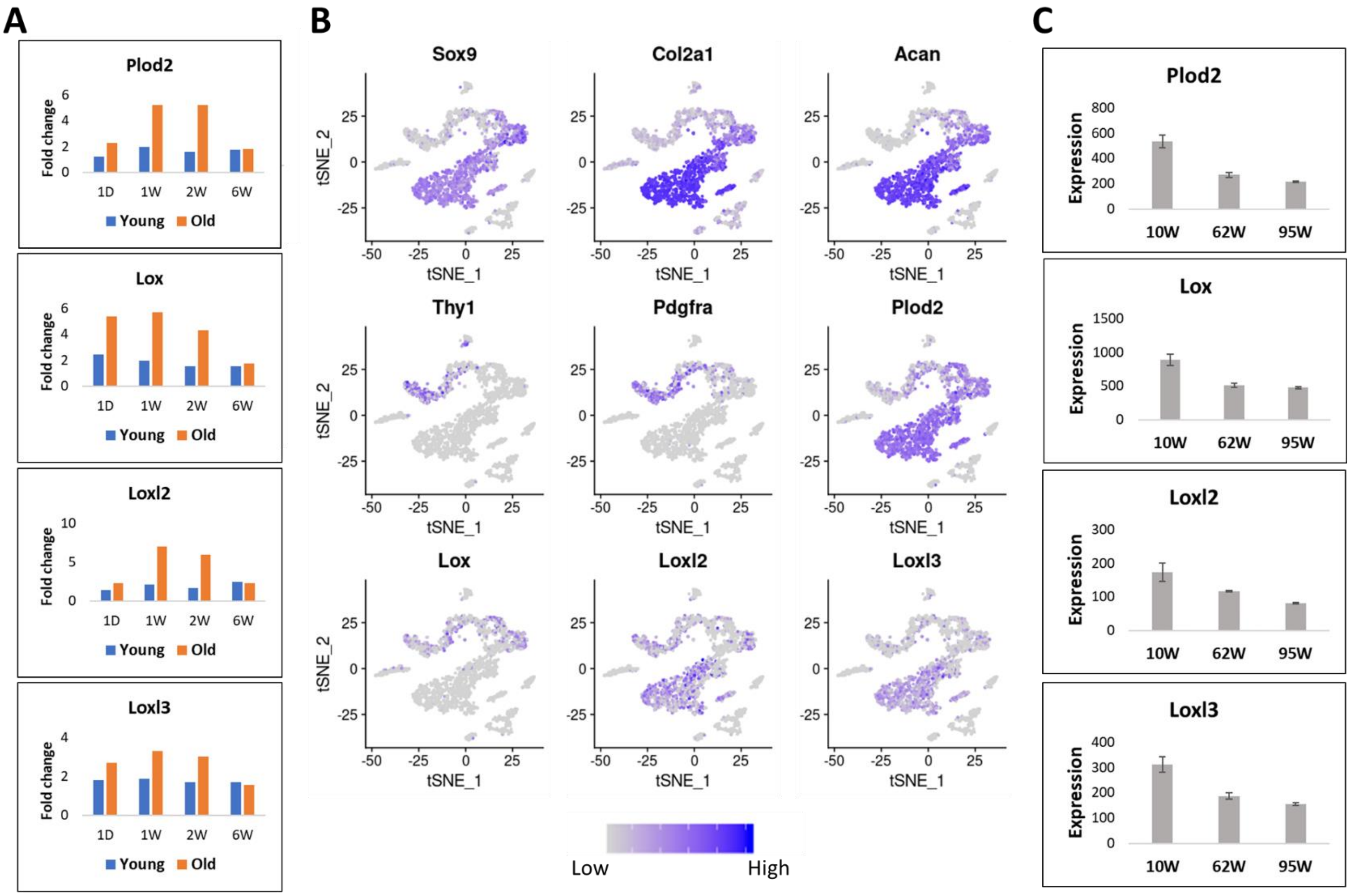

Supplement: Supplementary file 1 [file ijms-21-00364-s001.zip › Supp.info/Supplementary Figures_Sebastian et al_2019.pdf]
